# Use of a demonstration project to evaluate viral survival in feed: Proof of concept

**DOI:** 10.1111/tbed.13682

**Published:** 2020-06-26

**Authors:** Scott Dee, Apoorva Shah, Roger Cochrane, Travis Clement, Aaron Singrey, Roy Edler, Gordon Spronk, Megan Niederwerder, Eric Nelson

**Affiliations:** ^1^ Pipestone Applied Research Pipestone Veterinary Services Pipestone MN USA; ^2^ SAM Nutrition Eden Prairie MN USA; ^3^ Department of Veterinary and Biomedical Sciences South Dakota State University Brookings SD USA; ^4^ Department of Diagnostic Medicine/Pathobiology College of Veterinary Medicine Kansas State, University Manhattan KS USA

**Keywords:** animal feed, demonstration project, proof of concept, swine viral diseases

## Abstract

In 2014, the hypothesis that feed ingredients could serve as vehicles for the transport and transmission of viral pathogens was proposed and evaluated by multiple investigators under laboratory conditions. In an attempt to validate these data, we used a demonstration project to test whether three significant viruses of swine could survive in feed ingredients under real‐world shipping conditions. Samples of soya bean meal (organic and conventional), lysine, choline and vitamin A were spiked with a mixture of PRRSV 174, PEDV and SVA and transported for 21 days in the trailer of a commercial transport vehicle, encompassing 14 states and 9,741 km. Samples were tested for viral genome and viability at the end of the transit period. Regarding viability, PRRSV, PEDV and SVA were all detected as infectious in bioassays following inoculation with both soy products. In addition, viable PRRSV and SVA were detected by bioassay pigs inoculated with samples of vitamin A, and infectious SVA was detected in pigs inoculated with samples of lysine and choline. These results provide further evidence that select viral pathogens of pigs can survive in certain feed ingredients during commercial transit.

## 1 INTRODUCTION

In 2014, feed and feed ingredients were proposed as vehicles for the survival, transport and transmission of porcine epidemic diarrhoea virus (PEDV) from China to the United States (Dee et al., 2014; Dee et al., 2015, 2016). Since those seminal experiments, this hypothesis has been validated by several investigators across multiple viruses, such as Seneca virus A (SVA), porcine reproductive and respiratory syndrome virus (PRRSV), classical swine fever virus (CSFV), pseudorabies virus (PRV) and African swine fever virus (ASFV) (Dee et al., 2018; Stoian et al., 2019). These studies have also repeatedly confirmed that certain feed ingredients, such as soy‐based products, appear to promote viral survival over time. (Dee et al., 2014; Dee et al., 2015, 2016, 2018; Stoian et al., 2019, 2020). While these data have been well received by the North American swine industry and the veterinary profession, a significant limitation of these projects has been that data were derived solely from models conducted under laboratory conditions, both inherently small in scale and artificial in nature.

To address these acknowledged limitations, we designed an experiment using an approach taken from the social sciences known as the demonstration project. A demonstration project is defined as a means of promoting innovations and disseminating best practice through the development and analysis of a live project, undertaken in natural settings that resemble the non‐experimental (real‐world) conditions (Rutman, 1974). This approach has been used to help build an evidence base to support industry improvements, as historically, lessons learned from demonstrations, through the rigours of scientific research, have resulted in large‐scale adoption and major shifts in aims, styles and resources (Rutman, 1974). Therefore, the purpose of this study was to design a ‘proof of concept demonstration project to validate data from laboratory studies and to evaluate viral survival in feed ingredients under more representative, ‘real‐world’ conditions. The study was based on the hypothesis that viral survival is ingredient‐dependent.

## 2 MATERIALS AND METHODS

### 2.1 Sample preparation

Viruses selected for this study included PRRSV‐174, PEDV and SVA, while ingredients included conventional soya bean meal, organic soya bean meal, choline chloride (60%, no corn cob carrier), lysine HCL (78.8% minimum lysine, no carrier) and vitamin A (1,000,000 IU with porcine‐coated gelatin) Dee et al., 2015, Dee et al., 2016, Dee et al., 2018, Stoian et al., 2019). As previously defined, conventional soya bean meal contained a low fat (1%–2%) and high protein (46%–47%) content, while the organic product had higher fat (6%–7%) and lower protein content (44%–45%) (Dee et al., 2016, 2018). Samples of each ingredient were obtained from local mills and were not irradiated prior to initiating the study. Four 30 g allotments of the five ingredients were weighed into individual 50‐ml mini‐bioreactor tubes with vented caps (Corning Inc.) for a total of 20 samples, providing four replicates per ingredient. For preparation of the viral inoculum to be used to spike ingredients, each virus was diluted in 100 ml minimum essential medium (MEM, Sigma‐Aldrich) to a concentration of 1 × 10^5^ TCID_50_/ml per virus and mixed together (3 viruses for a total of 300 ml) followed by an addition of 200 ml MEM, to bring the total volume to 500 ml. Each of the 20 samples was individually spiked with a 2 ml aliquot from the viral mixture. Inoculums were injected directly into the centre of each 30 g ingredient sample using a 3‐ml syringe with an 18‐gauge, 3.81‐cm needle. In addition to the 20 spiked samples, two positive controls (stock virus mixture in the tube in the absence of feed), two negative controls (30 g conventional soya bean meal, no virus) and one contamination control (empty tube, no feed, no virus) were included in the design.

### 2.2 Details of demonstration project

#### 2.2.1 Transport vehicle

To house the samples during transit, a commercial dock, truck and trailer (7.9 m long × 2.4 m high × 2.4 m wide) were used. Samples were stored in a cardboard container and fastened to the trailer floor in the right rear corner using duct tape, with no climate control provided, thereby allowing samples full access to the changing environment during the entire transit period. During the project, no other cargo was included in the trailer and no deliveries occurred during the route. To record environmental conditions experienced during the trip, a data logger (RC‐51H, ELITech, Paris, FR) was placed alongside the samples and temperature and per cent relative humidity (% RH) were recorded every 15 min during each day in transit. In addition, a GPS system within the transport vehicle was used to track location, time in transit and distance travelled.

### 2.3 Details of travel plan

**FIGURE 1:**
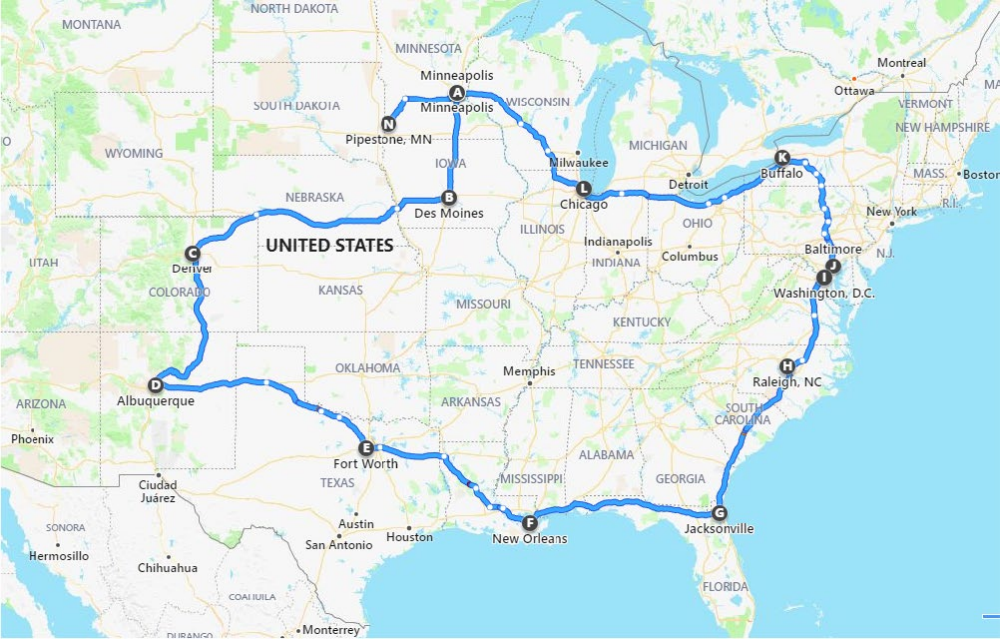
Map displaying the planned route of travel during the demonstration project period, providing a wide diversity in environments for ingredients and viruses

In order to expose ingredients and viruses to a wide variety of environmental conditions, it was planned to initiate transport of samples in Minneapolis, Minnesota, travel south to Des Moines, IA (overnight stay), to Denver, Colorado (overnight stay), to Albuquerque, New Mexico (overnight stay) to Fort Worth, Texas (overnight stay), and then to New Orleans, Louisiana (overnight stay). Travel would then continue along the gulf coast across the states of Mississippi, Alabama and Georgia into Jacksonville, Florida (overnight stay), and proceed up the eastern seaboard to Raleigh, North Carolina (overnight stay), to Washington, DC to Baltimore, Maryland (overnight stay), returning to the Midwest via Buffalo, New York (overnight stay), to Chicago, Illinois (overnight stay), to Minneapolis, Minnesota, and then finally to Pipestone, Minnesota, where samples were retrieved and made available for testing. Figure 1 provides a visual summary of the route planned.

### 2.4 Diagnostic testing

Following completion of the transport period, samples were evaluated for the presence of viral RNA by PCR and for viability by swine bioassay. For PCR, samples were tested at the South Dakota State University Animal Disease Research and Diagnostic Laboratory (SDSU ADRDL) using published methods (Dee et al., 2014; Dee et al., 2015, 2016).. For bioassay, pigs were housed in the Pipestone Applied Research biosafety level 2 facility in accordance with the institutional animal care and use guidelines observed by the investigators’ ethical review board (Pipestone Applied Research IACUC trial number 2020‐02). The design of the bioassay included 25 three‐week‐old pigs, originating from a farm known to be naïve for PRRSV, PEDV and SVA which were housed in a single room. Pigs were penned according to ingredient (5 pens, 4 pigs per pen, 20 total pigs) and 5 additional pigs, designated as controls were housed in individual pens (2 positive control pigs in a pen and the 3 negative control pigs in a pen). Pen dividers and empty pen spaces between animal groups were used to eliminate nose‐to‐nose contact and minimize indirect transmission between pens. For preparation of the bioassay inoculum, each 30 g sample from the five feed ingredients was transferred to separate 250‐ml conical tubes, followed by the addition of 60 ml of sterile saline. Each sample was then homogenized and centrifuged 4000 *g* for 10 min, with supernatant decanted into a clean 50‐ml tube and recentrifuged at 4000 *g* for 10 min. Supernatant was then decanted into 10‐ml tubes and frozen at −80°C, in preparation for inoculation. All pigs were then inoculated with a 2 ml sample via the intramuscular route 2 ml via the oral route. Rectal swabs and blood samples were then collected at days 0, 7, and 14 post‐inoculation and submitted to the SDSU ADRDL for analysis.

### 2.5 Data analysis

Temperature and % RH data collected during the transport period were analysed using descriptive statistics.

## 3 RESULTS AND DISCUSSION

### 3.1 Details of project period

The demonstration project took place over a period of 21 days from 20 February 2020 to 11 March 2020. It involved 107 hr in transit and covered 14 states for a total of 9,741 km (Figure 1). Descriptive statistic of the temperature and % RH data collected during the study period is provided in Table 1.

**Table 1:** Descriptive summary of temperature and % RH data collected every 15 min during the 21‐day transport period

	Temperature (°C)	Relative humidity (%)
# data points	2,140	2,140
Mean	9.3	35.7
Maximum	28	75.3
Minimum	−17	10.4
Median	9.5	34.4
Standard deviation	8.2	10.9
*SEM*	0.18	0.24
95% CI upper mean	9.6	36
95% CI lower mean	8.9	35

### 3.2 Presence of viral nucleic acid in feed

**FIGURE 2:**
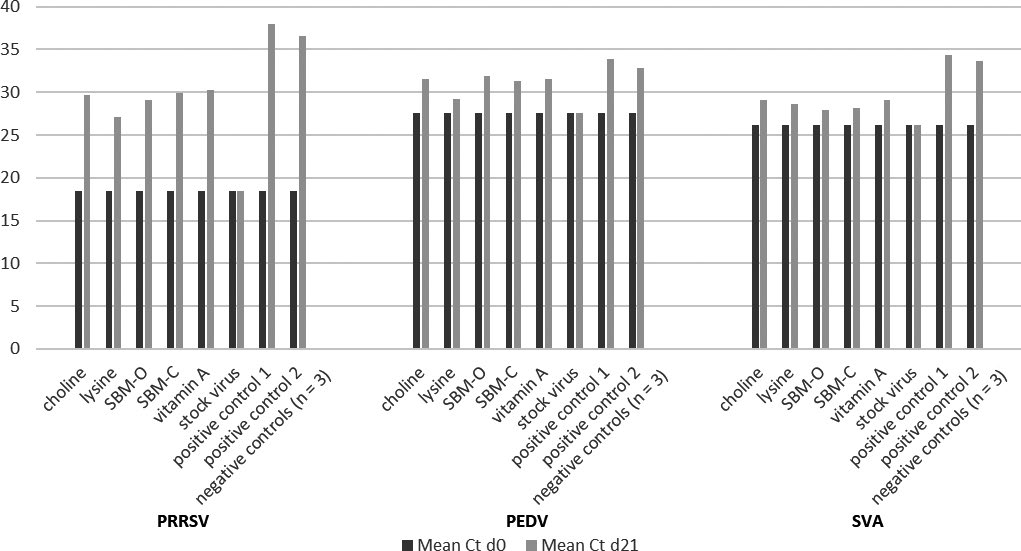
Summary of mean *C*_t_ values by virus and ingredient on day 0 and day 21 post‐inoculation, along with positive and negative controls. PCR‐negative samples, such as negative controls, were given a value of ‘0’. Note the consistent stability of SVA and PEDV, independent of ingredient, as compared to PRRSV 174

Viral load in stock virus preparations (no feed) at day 0 post‐inoculation and at day 21 post‐inoculation across the three viruses mixed with the five ingredients are summarized in Figure 2. While consistent stability of all three viral genomes across all five ingredients throughout the transport period was observed, degradation was numerically greater with PRRSV 174. In addition, PCR analysis indicated the presence of viral RNA in positive control samples with no evidence of RNA in negative controls.

### 3.3 Viability assessment

Prior to inoculation, all pigs were confirmed to be naïve to all three viruses via serum samples and rectal swabs collected on day 0. In contrast, on day 7 post‐challenge, PRRSV, PEDV and SVA RNA were detected by PCR in serum samples from three of the four bioassay pigs in the organic soya bean meal group and from four of the four bioassay pigs in the conventional soya bean meal group. PRRSV RNA (one of four pigs) and SVA RNA (three of four pigs) were detected in sera from animals inoculated with samples of vitamin A, while only SVA RNA was detected in animals inoculated with samples of lysine (four of four pigs) and choline (one of four pigs), respectively. In addition, clinical signs suggestive of PRRSV (dyspnoea, hyperthermia), PEDV (diarrhoea) and SVA (lameness) were observed in infected animals. In contrast, viral nucleic acid was not detected in any animals inoculated with positive control samples (stock virus, no feed ingredient) or negative controls (conventional soya bean meal, no virus) and these animals were also clinically normal. These results were confirmed on day 14 post‐inoculation and are summarized in Table 2.

**Table 2:** Summary of viral viability by ingredient type according to swine bioassay

	Conventional soya bean meal	Organic soya bean meal	Lysine	Choline	Vitamin A
PRRSV	4 of 4 pigs (+)^a^	3 of 4 pigs (+)	0 of 4 pigs (+)	0 of 4 pigs (+)	0 of 4 pigs (+)
PEDV	3 of 4 pigs (+)	3 of 4 pigs (+)	0 of 4 pigs (+)	0 of 4 pigs (+)	0 of 4 pigs (+)
SVA	4 of 4 pigs (+)	4 of 4 pigs (+)	4 of 4 pigs (+)	0 of 4 pigs (+)	3 of 4 pigs (+)
(+) controls^b^	0 of 2 pigs (+)	0 of 2 pigs (+)	0 of 2 pigs (+)	0 of 2 pigs (+)	0 of 2 pigs (+)
(−) controls^c^	0 of 3 pigs (+)	0 of 3 pigs (+)	0 of 3 pigs (+)	0 of 3 pigs (+)	0 of 3 pigs (+)

^a^ A determination of (+) was based on a positive PCR test from a blood sample (PRRSV or SVA) or a positive PCR test from a rectal swab (PEDV or SVA) following inoculation with a designated feed ingredient.

^b^ Positive control pigs were inoculated with a stock virus mix of PEDV, PRRSV and SVA (no feed matrix) following conclusion of the transport period.

^c^ Negative control pigs were inoculated with conventional soya bean meal (no virus) following conclusion of the transport period.

The purpose of this study was to use a demonstration project approach to further evaluate viral survival in feed ingredients under conditions of an actual transcontinental shipping event. Not only do the results validate previously published laboratory data, but they also further demonstrate the ability of viruses to survive in feed ingredients under robust conditions including long distances (9,741 km), extended time periods (21 days) and diverse environments (14 US states). The project further validated the ability of SVA, a picornavirus and accepted surrogate for foot‐and‐mouth disease virus, to survive in multiple feed ingredients providing additional evidence in support of the protective effect of soy‐based products (4). In addition, the inability of all three viruses to survive in stock preparations consisting of minimal essential medium in the absence of a feed matrix suggests that certain feed ingredients may have the ability to shield select viruses from environment stressors (Dee et al., 2016, 2018).

Despite these advancements, as with all experiments this study had its share of acknowledged strengths and limitations. Strengths included the novelty of the demonstration project approach, the use of multiple replicates per ingredient, the inclusion of negative controls to confirm that cross‐contamination did not occur and a novel study design that included real‐world shipping conditions. In addition, biosecurity was maximized during transit, since no other products were included in the trailer and no stops were made at agricultural sites, only hotels. Prior to initiation, this project was discussed with state animal health officials and feedback was positive regarding the safety of the project with these practices in place. In contrast, limitations of the study included the use of a single viral concentration to inoculate the ingredient samples. While this concentration was based on previously published data on PEDV in feed (Dee et al., 2014), unfortunately information regarding viral load in actual feed samples is limited at this time, as testing of feed ingredients for foreign animal diseases is not currently allowed in the United States. Another significant limitation was the use of a small sample size, along with small quantities (30 g) of feed ingredients spiked with large volumes of liquid inoculum. While small quantities were used to minimize the risk of false‐negative results, studies are underway to repeat this project using larger volumes (1,000 kg totes) of ingredients inoculated with proportionately representative volumes of liquid inoculum. Finally, while this study provided information on the survival of viruses in feed, it purposefully did not address transmission of viruses through feed or infectivity using natural feeding behaviour.

In closing, despite these acknowledged limitations, this study did demonstrate that three significant viral pathogens of pigs could survive in select feed ingredients during commercial transport, involving diverse environmental conditions and realistic transit periods. It is hoped that the information derived from this study will help to unify opinions across the swine industry, the veterinary profession and governmental agencies regarding the risk of feed. Until we are united, we cannot make progress at the continental level, and until that time, we all are at risk.

## CONFLICT OF INTEREST

The authors declare no conflict of interest.

## ETHICAL APPROVAL

Animals in this study were managed in accordance with the institutional animal care and use guidelines observed by the investigators’ ethical review board, Pipestone Applied Research IACUC, trial number 2020‐02.

## Data Availability

All data from this study have been disclosed.
